# Peroneal Tendon Tears: Four Simple-to-Complex Cases

**DOI:** 10.7759/cureus.73787

**Published:** 2024-11-15

**Authors:** Zhi Hao Tang, Keen Wai Chong

**Affiliations:** 1 Orthopaedic Surgery, Khoo Teck Puat Hospital, Singapore, SGP; 2 Orthopaedics, Bjios Orthopaedics, Singapore, SGP

**Keywords:** cavovarus deformity, peroneal ruptures, peroneal tears, tendon repair, tendon transfer, tenodesis

## Abstract

Peroneal tears are an important cause of lateral ankle pain and are often missed. Peroneal tears can present in different combinations requiring different surgical strategies. If the tears are symptomatic in patients in whom conservative treatment has failed, surgery is an option. We present the various types of surgical management of four patients, each with a different tear combination of the peroneal tendons. The first patient presented with a longitudinal split of the peroneal brevis tendon, which was repaired. The second patient had a tear of the peroneal longus tendon with a significant gap, while his peroneal brevis tendon was intact. His peroneal longus was tenodesed to the intact peroneal brevis.

The third patient had ruptures of both his peroneal brevis and longus tendons with significant gaps. There was only a small peroneal brevis remnant left. The patient also had a cavovarus deformity of the same foot. His flexor hallucis longus tendon was harvested, routed, and sutured to the remnant peroneal brevis tendon. A lateralising calcaneal osteotomy and a dorsiflexion closing wedge osteotomy of his first metatarsal bone were also performed. The last patient had ruptures of both peroneal tendons with no remnant tendon remaining for repair. His anterior tibialis tendon was transferred from its insertion to his cuboid. A lateralising calcaneal osteotomy was performed, and an ankle-spanning external fixator was applied. A high index of suspicion for peroneal tears in lateral-sided ankle pain must be maintained. Peroneal tears can present in various combinations, with each combination requiring a different surgical treatment.

## Introduction

Peroneal pathology has been recognised as an important cause of posterolateral ankle pain. This pathology includes peroneal tendon tears, subluxation/dislocation, and tendinosis as well as tenosynovitis. Peroneal tendon tears in particular can be divided into acute and chronic. Acute injuries usually occur after a traumatic event, while chronic ones usually are attritional in nature, with acute tears being less common. There is currently no consensus on the specific timeframe in which the injury can be considered acute or chronic. Peroneal tears with instability are often the result of sports injuries. Hindfoot varus and lateral ankle instability can also increase the tension in the peroneal tendons and predispose them to tears [1]. Patients may present with a chronic condition if the initial diagnosis is missed [2]. Injuries to these tendons can affect the function of the foot [3]. This can cause persistent lateral ankle pain and functional instability if left untreated, as peroneal tendons act as primary evertors of the foot.

Overall incidence of peroneal tears is higher than previously thought. Only 60% of peroneal tendon pathology was diagnosed on the first clinical assessment [4]. A high index of suspicion for peroneal tears should be maintained when a clinician is treating patients with lateral-sided ankle pain. Surgical treatment can be considered in symptomatic patients. However, the various combinations of the number of tendons involved, tears or ruptures, types of tears (acute or chronic), any presence of deformity, as well as the quality and quantity of remnant tendon substance left, presents a management challenge for the treating clinician. So far there is no optimal management algorithm available. In this paper, we present four patients with various combinations of peroneal tears and their different types of surgical management, each tailored to each individual patient.

## Case presentation

Case one

The first patient is a 24-year-old female who presented with acute lateral ankle pain after an inversion injury to her right ankle, which was worse with walking on uneven ground. Clinically there was tenderness along the peroneal tubercle, associated with swelling. The pain was worse with resisted eversion. Her hindfoot alignment was within normal limits with supple ankle and subtalar joints. After the initial clinical assessment, she was diagnosed to have a peroneal tendon tear. As she was able to resist eversion, this was not deemed to be a rupture, and she was treated conservatively with rest, activity modification, and a walker boot for six weeks. After six weeks, she had persistent pain and hence a magnetic resonance imaging (MRI ) scan was ordered.

**Figure 1:**
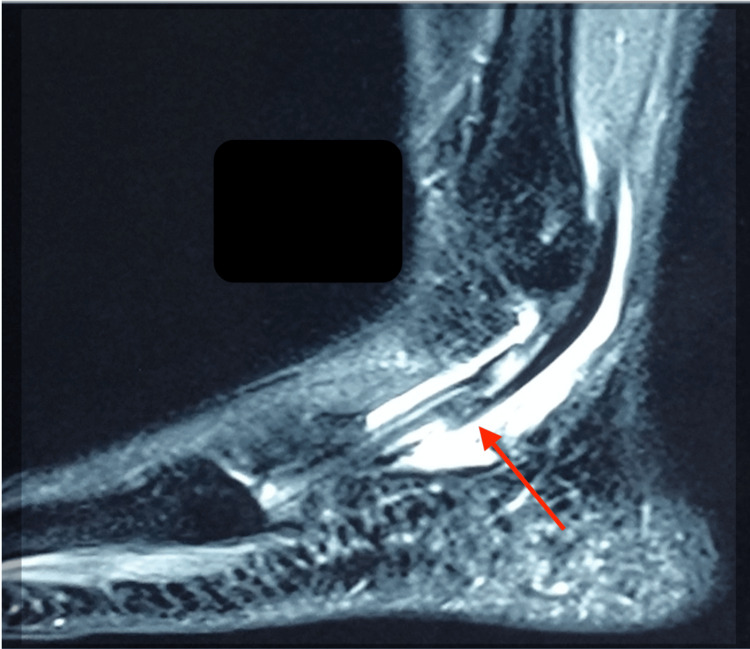
MRI sagittal view of the patient’s right ankle showing the longitudinal split (red arrow) in the peroneal brevis with fluid in the sheath.

**Figure 2:**
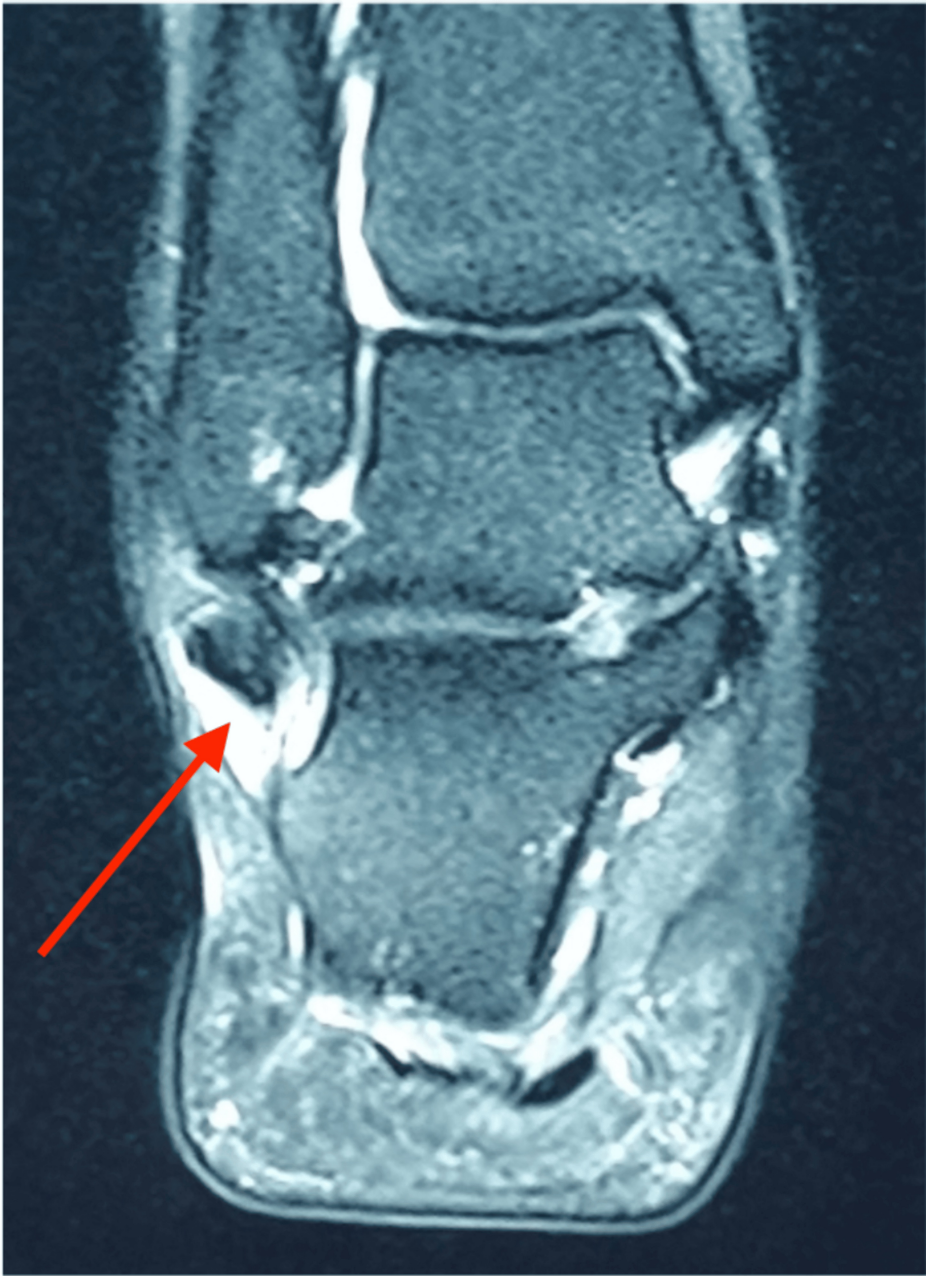
MRI coronal view of the patient‘s right ankle showing the distended fluid sheath (red arrow) of the peroneal brevis tendon.

Figures 1, 2 show the MRI scans of the patient’s right ankle, showing a longitudinal split in the peroneal brevis with intra-sheath fluid. 

**Figure 3:**
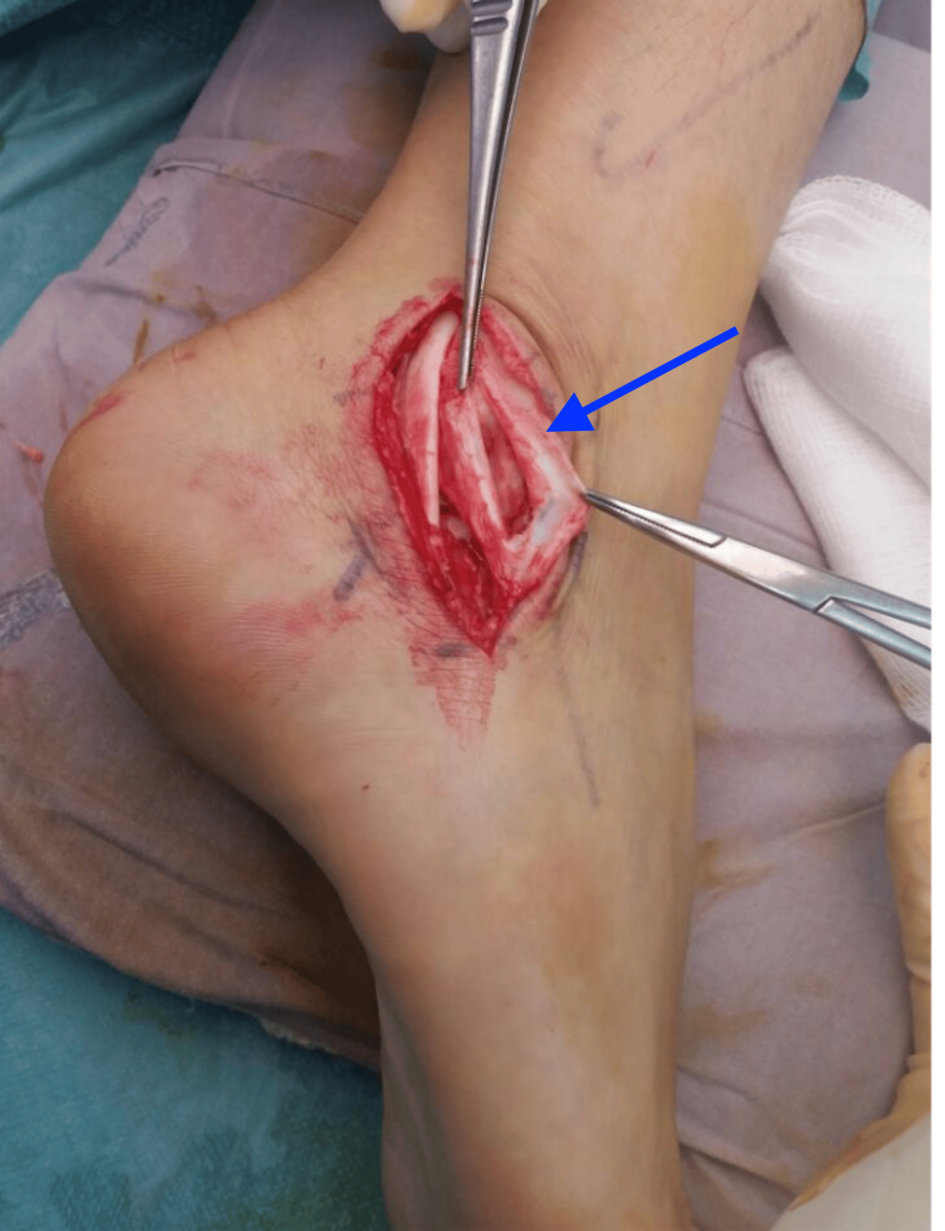
Intraoperative photograph showing a longitudinal split in the patient’s peroneal brevis tendon (blue arrow).

**Figure 4:**
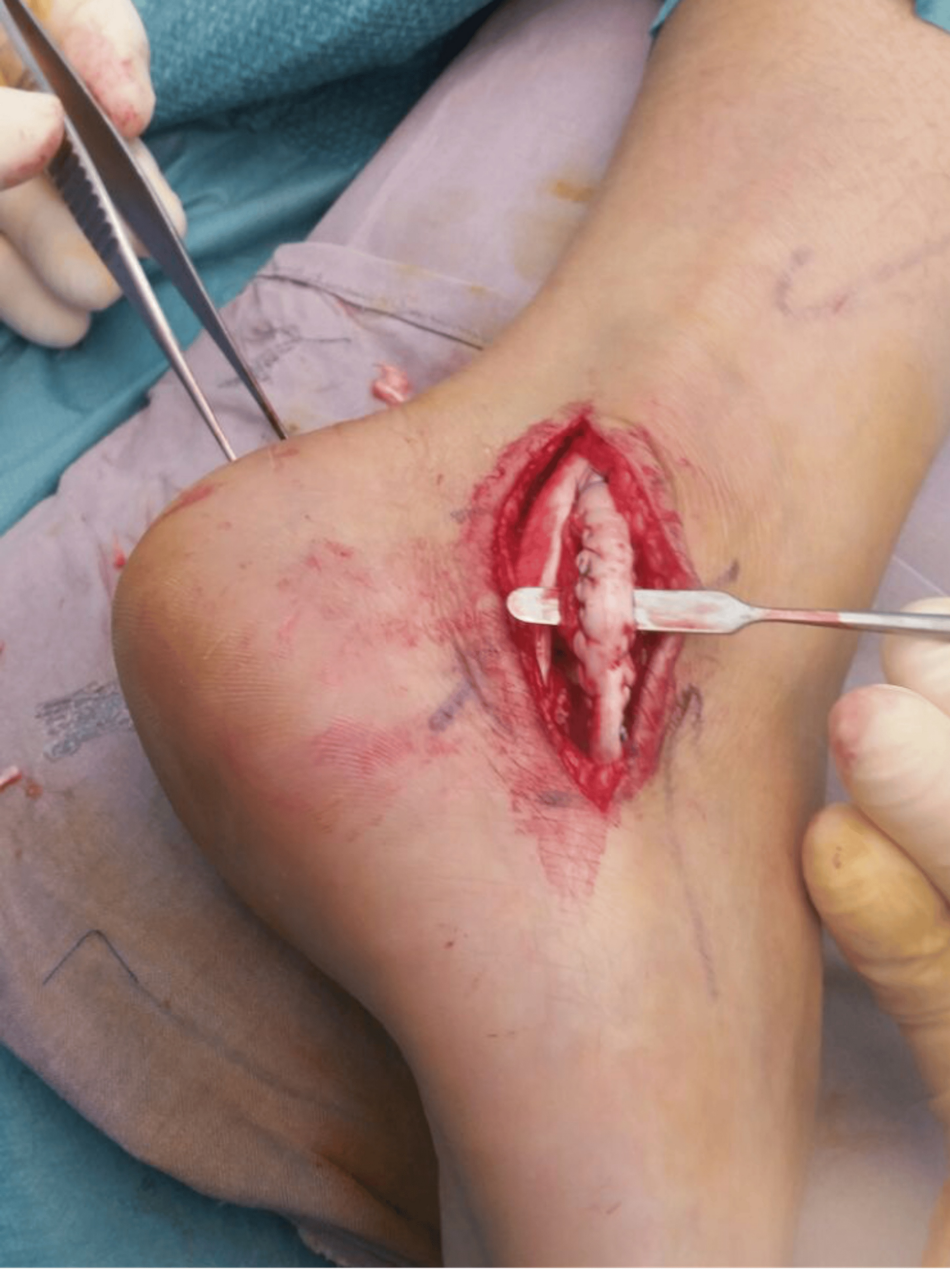
Intraoperative photograph showing the repaired peroneal brevis tendon.

Intra-operatively, the longitudinal split of the peroneal brevis was confirmed. The peroneal longus tendon was intact. The frayed edges of the split in the tendon were debrided, and the split was then repaired with a 3-0 non-cutting Prolene stitch (Ethicon Inc., Raritan, New Jersey, United States). The peroneal tubercle was also removed until a flat surface was obtained and the inferior peroneal retinaculum was released. Figure 3 shows the intraoperative photograph of the split peroneal brevis tendon, while Figure 4 depicts the repaired peroneal brevis tendon. 

Post-operatively, the patient was fitted with a walker boot and allowed to bear weight on the boot for six weeks. Skin stitches were removed at two weeks post-surgery. Thereafter, she was started on physiotherapy. At three months post-surgery, she was pain-free, could resume activities of daily living, and was able to start jogging on the treadmill. 

Case two

The patient was a 63-year-old male who similarly presented with an acute inversion injury to his left ankle while running. He had complained of persistent posterolateral ankle pain. On clinical examination, there was tenderness and swelling along the peroneal tendons near the peroneal tubercle. His ankle and subtalar joints were supple. The hindfoot alignment was neutral. The patient was initially seen by a fellow orthopaedic surgeon who had treated him conservatively with a walker boot. Four months after his injury, there was no relief of pain, and he was still unable to go back to running. An MRI scan of his left ankle was ordered by the initial treating orthopaedic surgeon.

**Figure 5:**
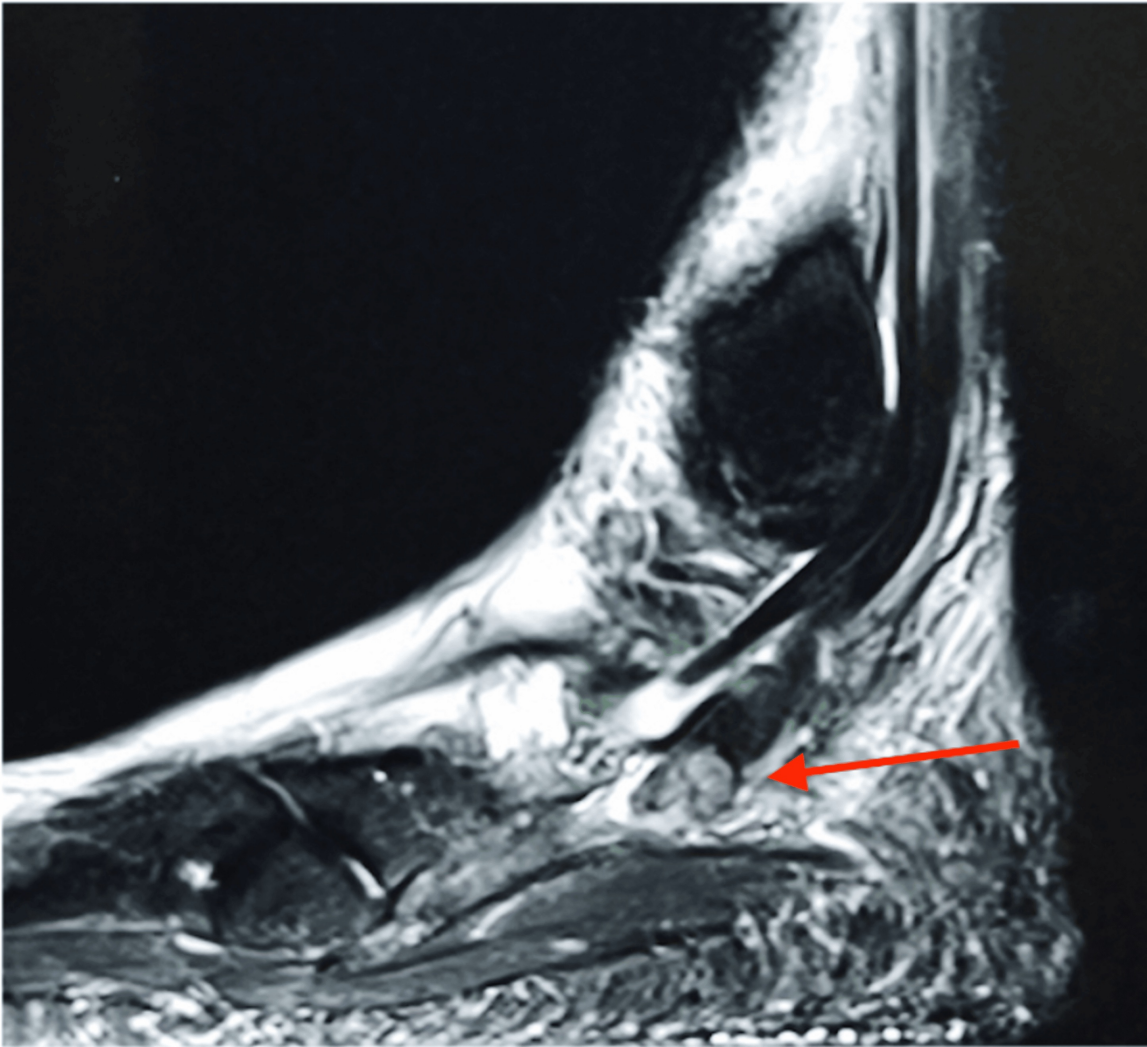
MRI sagittal image of the patient’s left ankle showing the proximal stump of the ruptured peroneal longus tendon (red arrow).

Figure 5 shows the MRI sagittal image of his left ankle showing the proximal stump of the ruptured peroneal longus. The distal stump was at the cuboid tunnel with a significant gap of 3.7 cm between the two stumps. The peroneal brevis tendon was intact.

**Figure 6:**
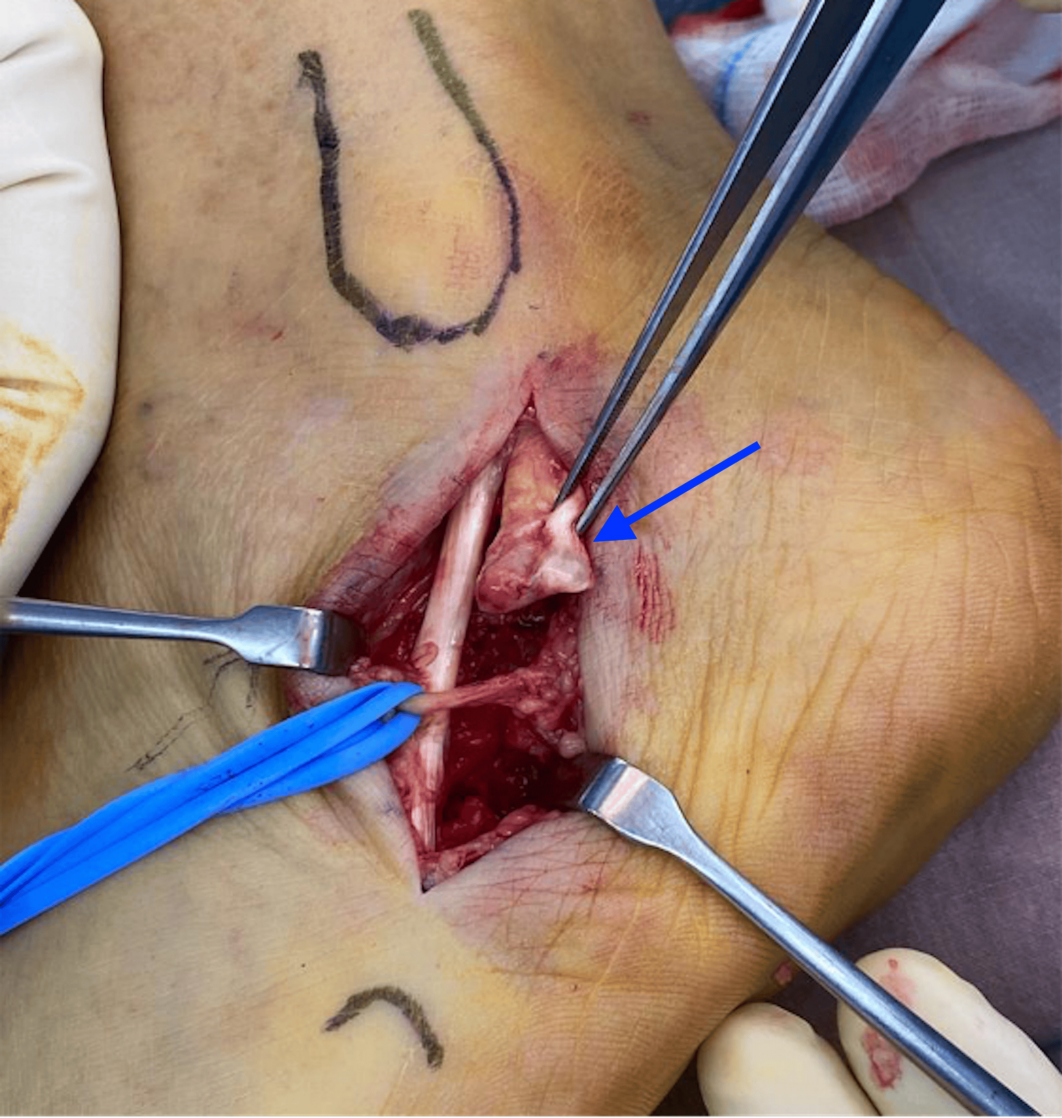
Intraoperative photograph of the lateral aspect of the patient’s left foot showing the proximal stump (blue arrow) of the peroneal longus rupture. The distal stump could not be found. The peroneal brevis tendon was intact. The sural nerve was safely retracted and protected with the vessel loop.

**Figure 7:**
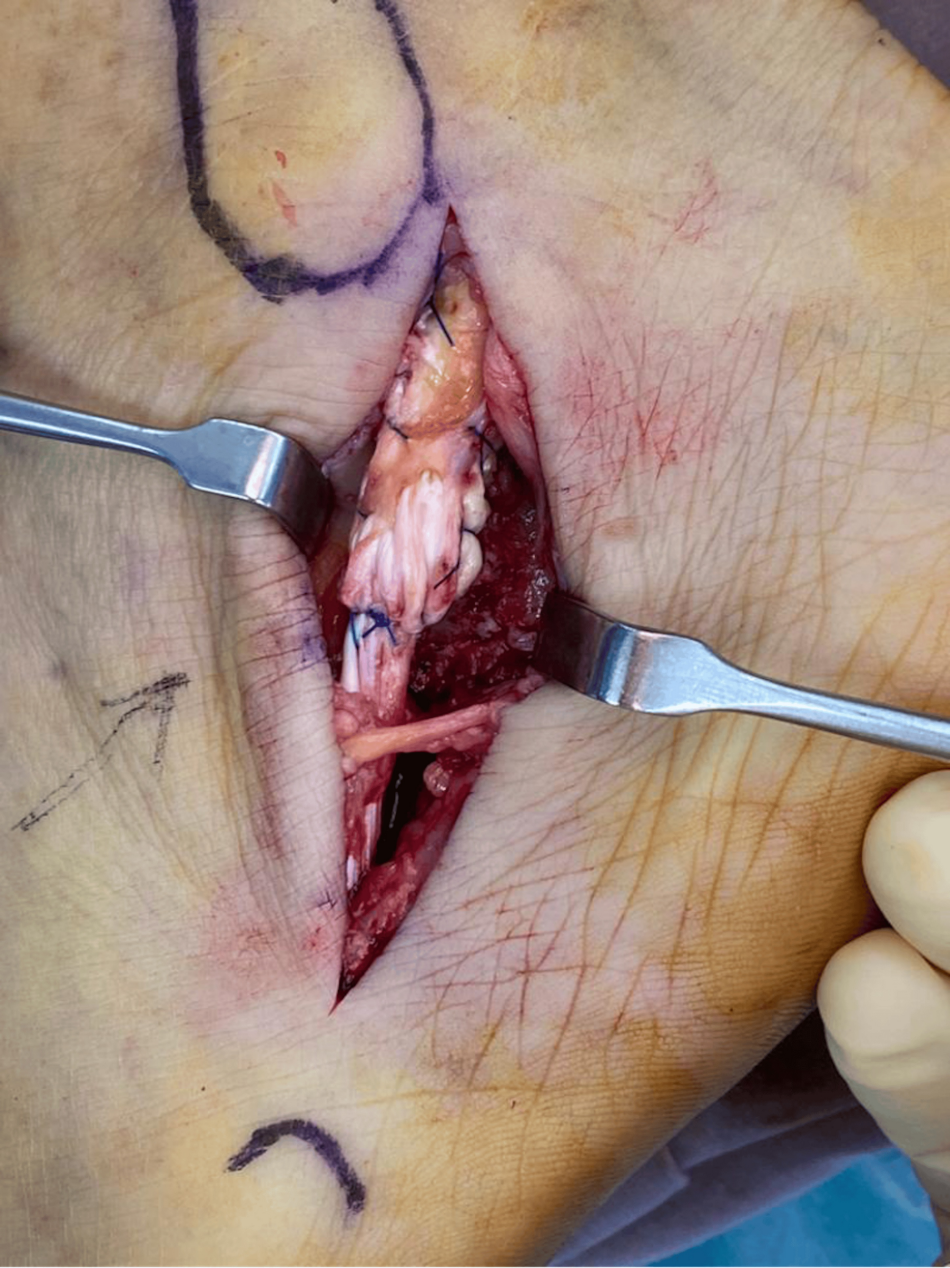
Intraoperative photo showing the proximal stump of the peroneal longus being tenodesed to the intact peroneal brevis tendon.

As the stump gap is too large for repair, the proximal stump of the peroneal longus was debrided and tenodesed to the peroneal brevis tendon with a 3-0 Prolene suture (Figures 6, 7).

Post-operatively, the patient was provided with a walker boot and allowed to bear weight with the boot for six weeks. Skin stitches were removed at two weeks post-surgery. The boot was removed at six weeks, and physiotherapy was started. He was able to return to running at six months post-surgery with the resolution of the pain.

Case three

**Figure 8:**
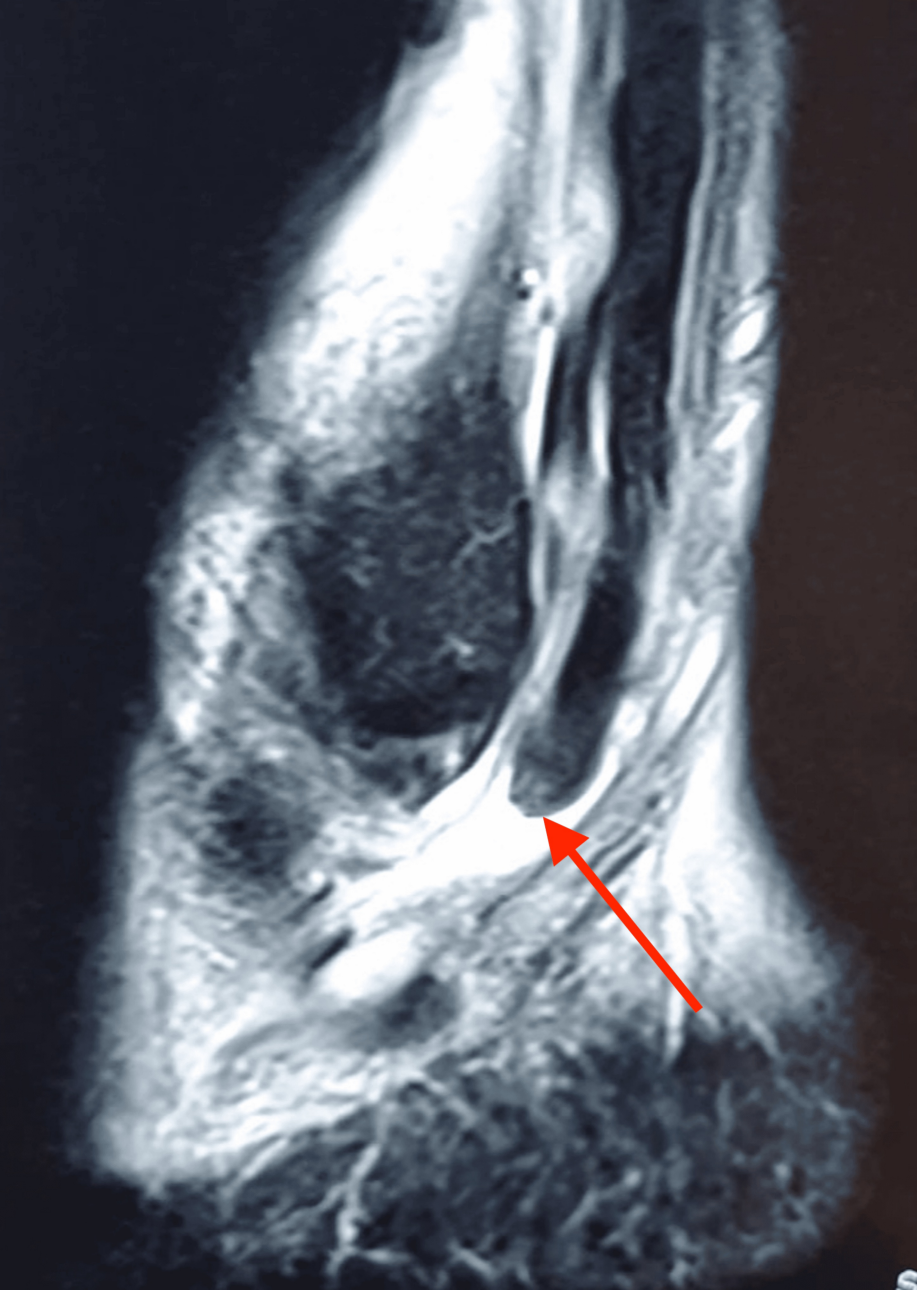
MRI sagittal image of the patient showing the proximal stumps (red arrow) of the ruptured peroneal brevis and longus tendons.

The third patient was a middle-aged general practitioner who was an avid badminton player who experienced lateral-sided left ankle pain while playing badminton. He had continued to play badminton despite the symptoms. He then self-treated his acute symptoms with anti-inflammatory medication and icing. After the acute pain improved, he did not seek further treatment and continued with his work and activities of daily living. However, he could not play badminton, felt weakness in his left ankle and found that his foot was getting malaligned. The pain recurred and became persistent and chronic. He then obtained an orthopaedic consult two months post-injury. On examination, there was swelling along the peroneal tendons and weakness in eversion strength. His hindfoot was in a varus alignment. The MRI of his left ankle revealed ruptures of both his peroneal brevis and longus tendons with only a small distal stump of the peroneal brevis remaining. Figure 8 shows the sagittal view of the MRI of his left ankle showing the proximal stumps of his ruptured peroneal brevis and longus tendons.

**Figure 9:**
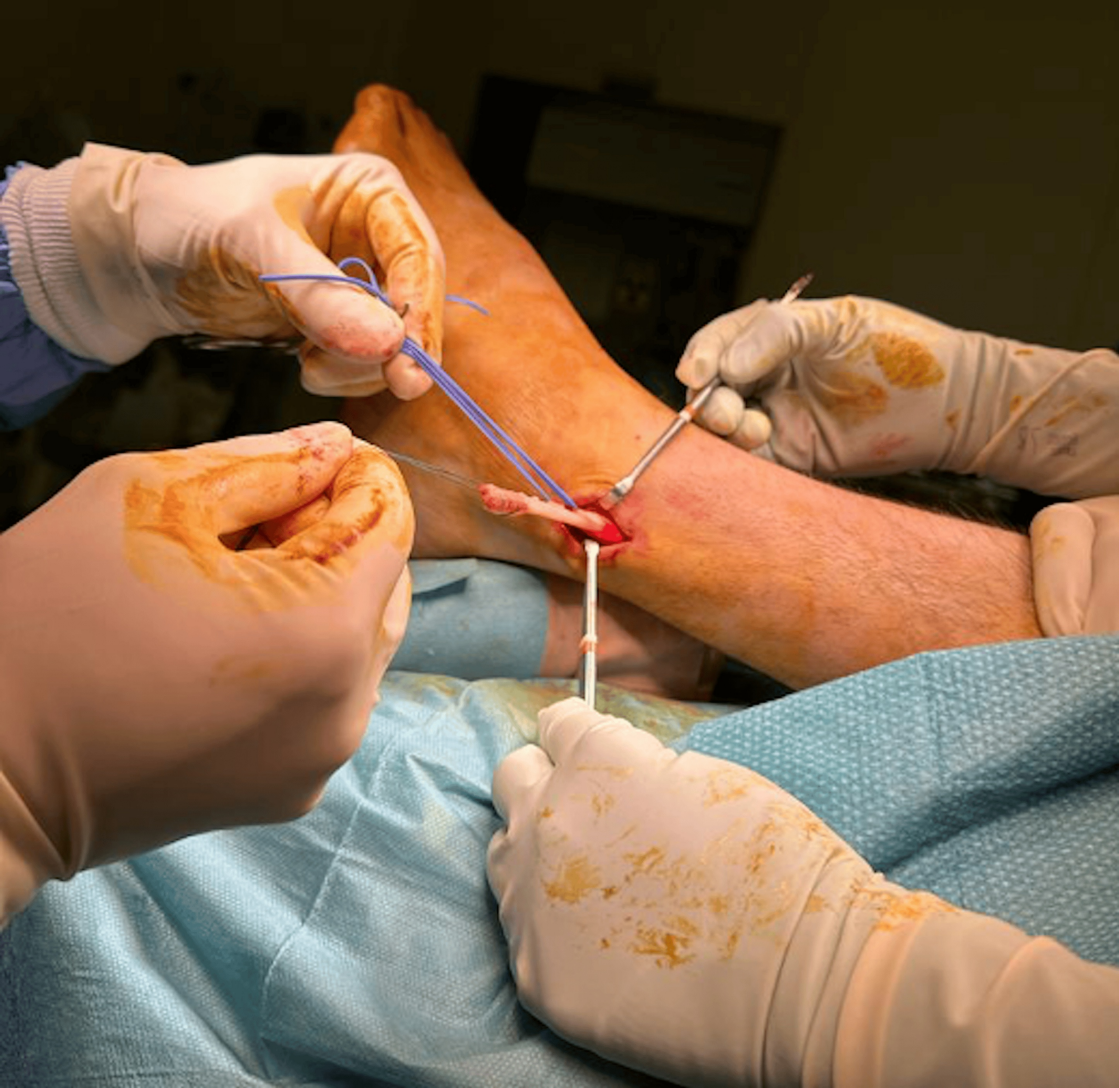
The flexor hallucis tendon delivered proximally, then posteriorly behind the tibia, and retrieved posterolaterally.

A surgical correction of the varus hindfoot was done using a calcaneal osteotomy via a lateral L-shaped extensile approach. The osteotomy was stabilised using two 6.5 mm headless compression screws inserted percutaneously. A dorsiflexion closing wedge of the first metatarsal osteotomy was performed as well to reduce the varus moment on the hindfoot. A small longitudinal incision was made over the plantar-medial aspect of the midfoot, and the flexor hallucis longus (FHL) tendon was identified just proximal to the knot of Henry and transected. The FHL then was delivered through another small incision over the posteromedial aspect tibia and routed posterior to the tibia and fibula to the lateral side through a lateral incision (Figure 9). The FHL tendon was then sutured to the remnant peroneal brevis stump with the ankle in neutral dorsiflexion and slight eversion. The patient was offered an ankle-spanning external fixator to protect the tendon transfer, but he declined. As an alternative, his foot was put in a plaster of Paris cast with the ankle in a neutral position for six weeks. The cast was then removed and he was started on physiotherapy. At eight months, he was able to return to non-competitive badminton pain-free. His hindfoot alignment remained neutral.

**Figure 10:**
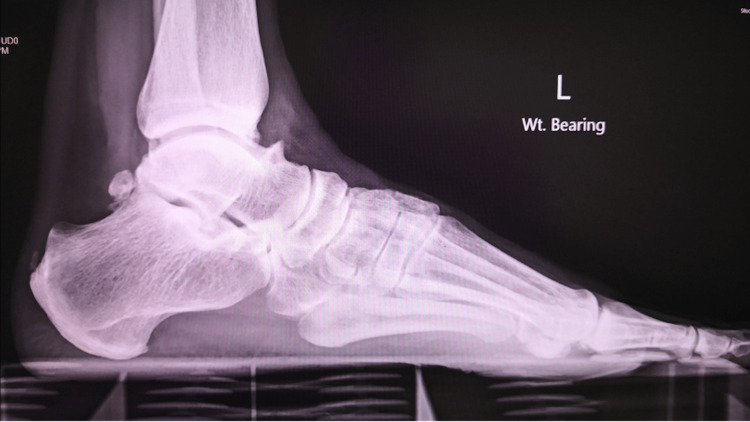
Pre-operative weight-bearing radiographs of the patient’s left foot.

**Figure 11:**
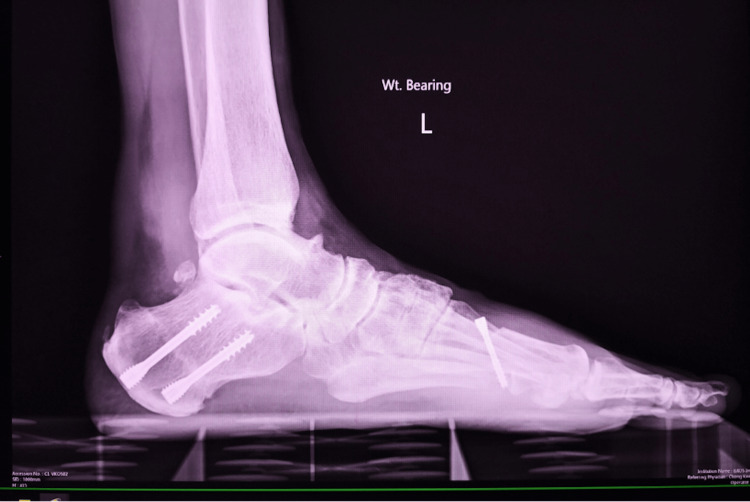
Post-operative weight-bearing radiographs of the patient’s left foot.

**Figure 12:**
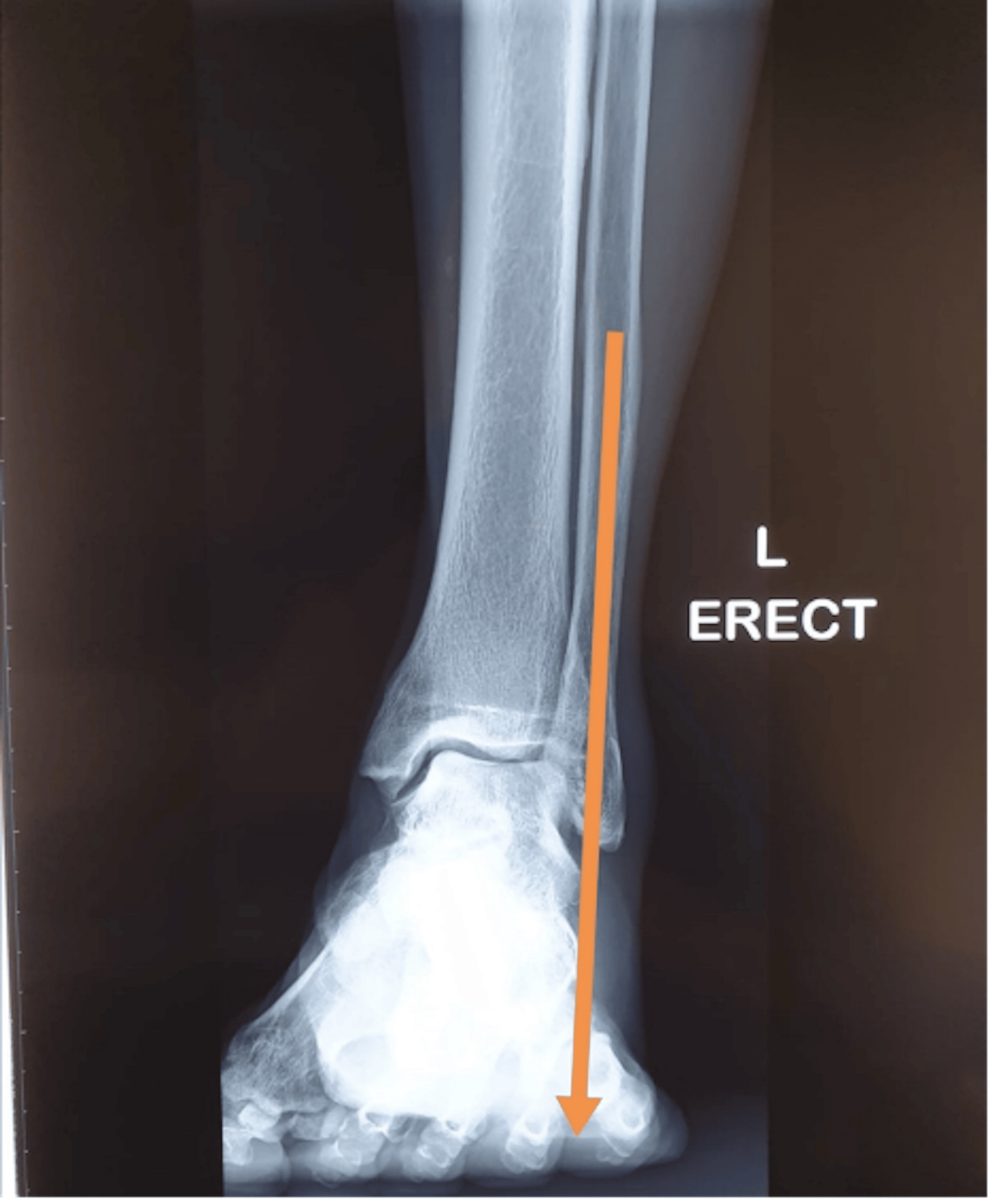
Pre-operative weight-bearing views of the patient’s left ankle.

**Figure 13:**
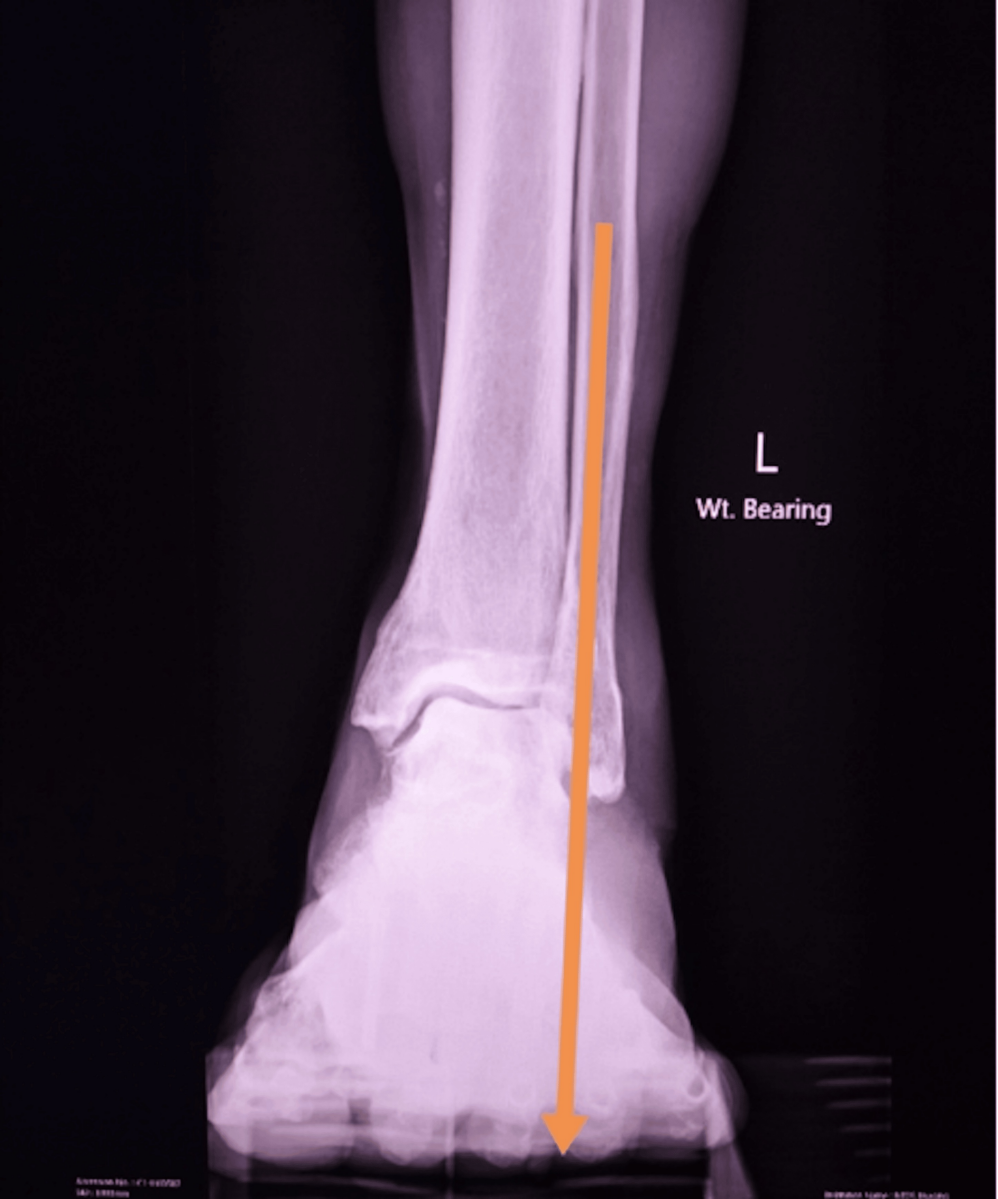
Post-operative weight-bearing views of the patient’s left ankle.

Figures 10, 11 show the weight-bearing lateral radiographs of the patient’s left foot pre- and post-surgery, respectively. Figures 12, 13 show the weight-bearing ankle views of the patient. The orange line depicts the longitudinal axis of the fibula shaft. The improvement in hindfoot alignment is assessed using the fibular-axis-calcaneal-offset method [5]. As shown, the calcaneal wall was medial to the fibular axis prior to the surgery (Figure 12). Post-hindfoot correction, the calcaneal wall was slightly lateral to the fibular axis (Figure 13).

Case four

**Figure 14:**
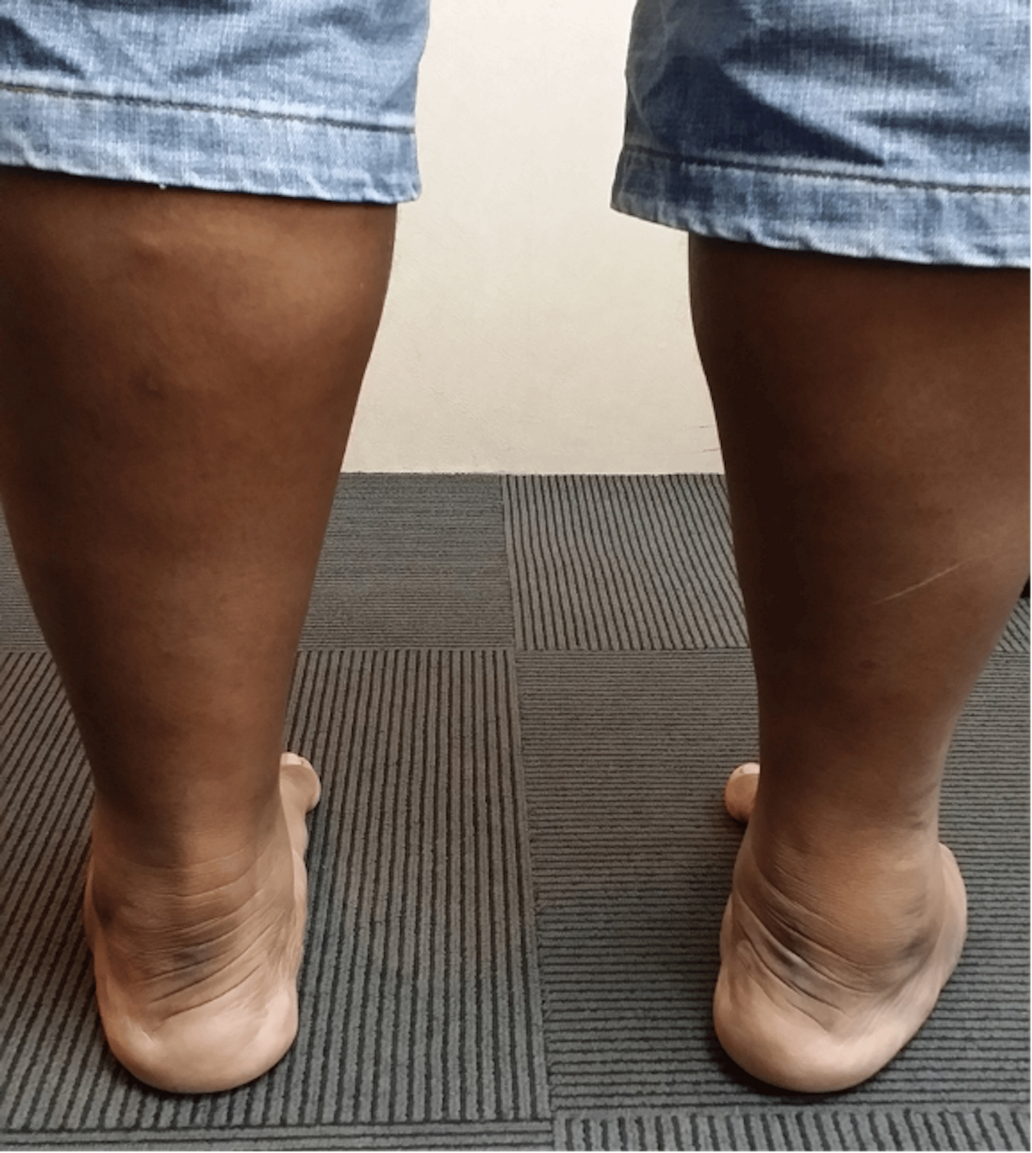
Clinical photograph of the patient’s varus hindfoot alignment.

**Figure 15:**
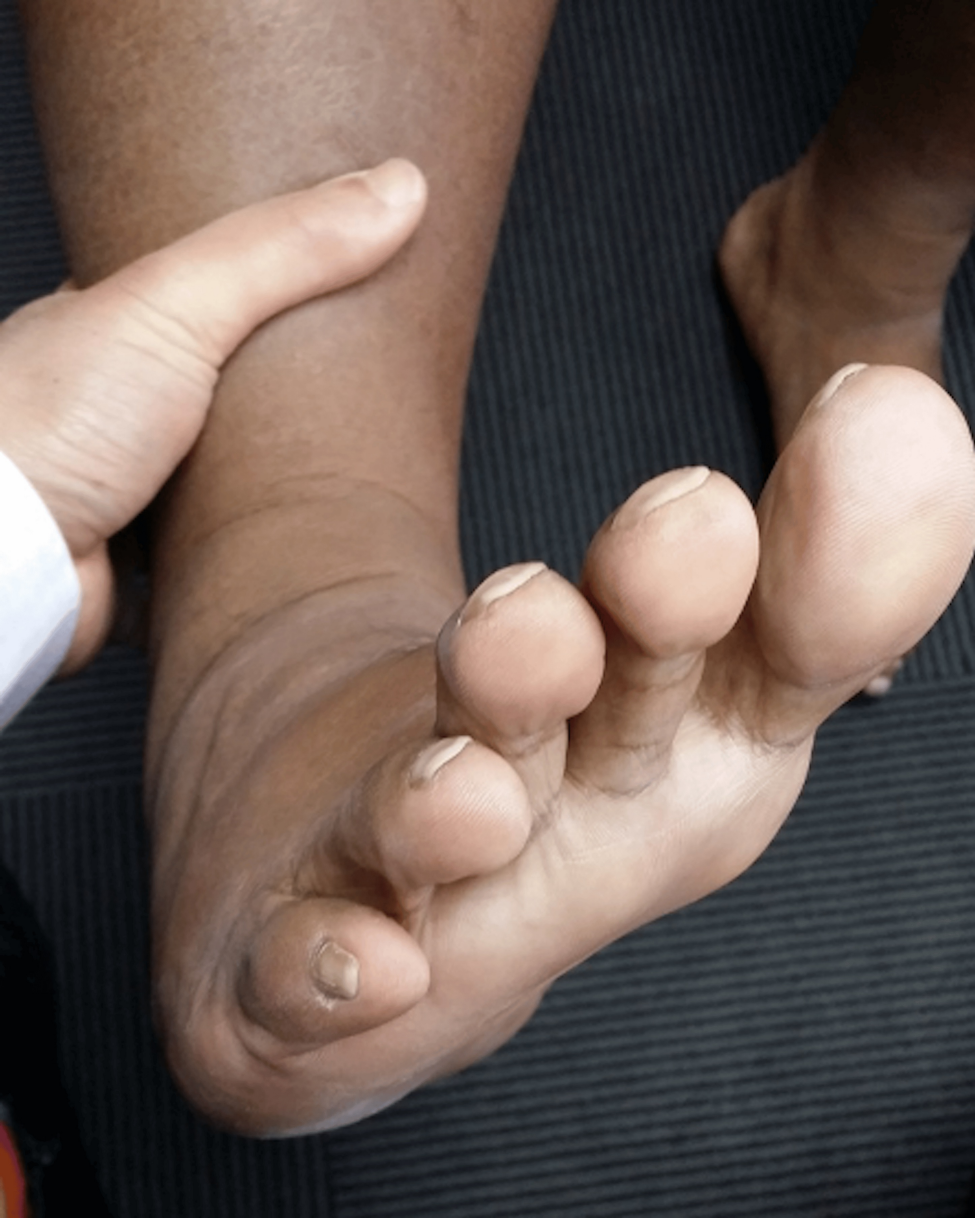
Clinical photograph of the patient’s right foot in an inverted resting position.

**Figure 16:**
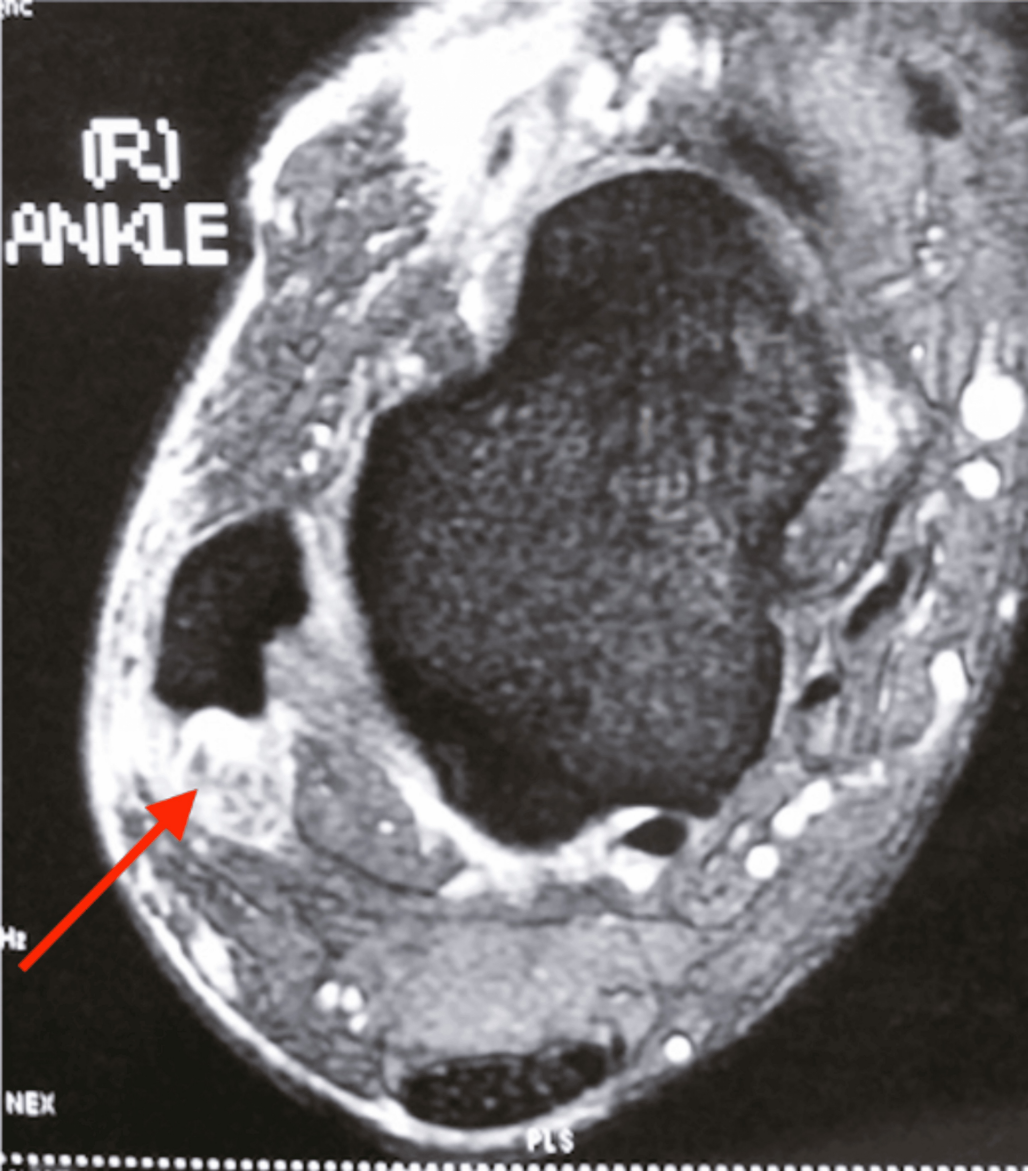
MRI axial cuts showing absent distal remnants of both peroneal tendons (red arrow).

**Figure 17:**
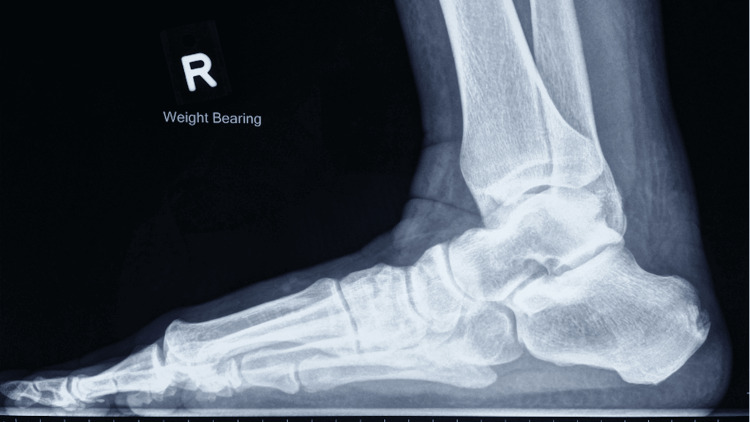
Pre-operative weight-bearing lateral radiographs of the patient’s right foot.

**Figure 18:**
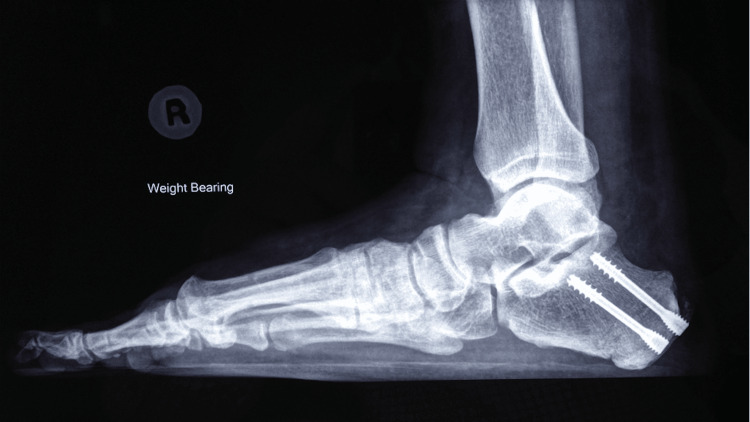
Post-operative weight-bearing lateral radiographs of the patient’s right foot.

This middle-aged patient presented with chronic right lateral heel pain. He had complained of a “floppy ankle” and frequent tripping. His ankle went into inversion when he was asked to dorsiflex his ankle. On clinical examination, eversion power was 0/5 and his hindfoot and forefoot were in inversion as shown in Figures 14, 15. As his symptoms had been there for more than one year, and were getting worse, an MRI scan was obtained. MRI showed ruptures of both the peroneal brevis and longus tendons with no distal remnant left on both tendons (Figure 16). A lateralising calcaneal osteotomy was performed via the same L-shaped lateral flap incision and stabilised with two 6.5 mm headless compression screws. Figures 17, 18 show the lateral weight-bearing views of the patient’s right foot pre- and post-surgery, respectively.

**Figure 19:**
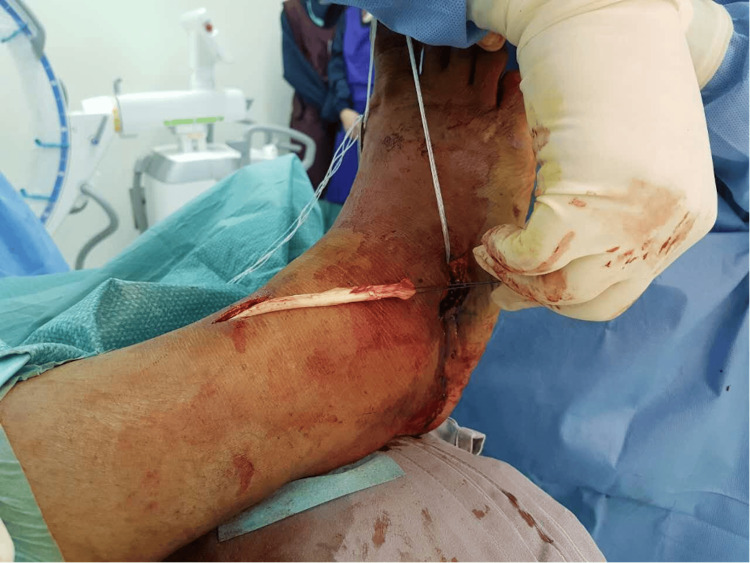
Intraoperative photograph of the patient’s harvested anterior tibialis tendon.

**Figure 20:**
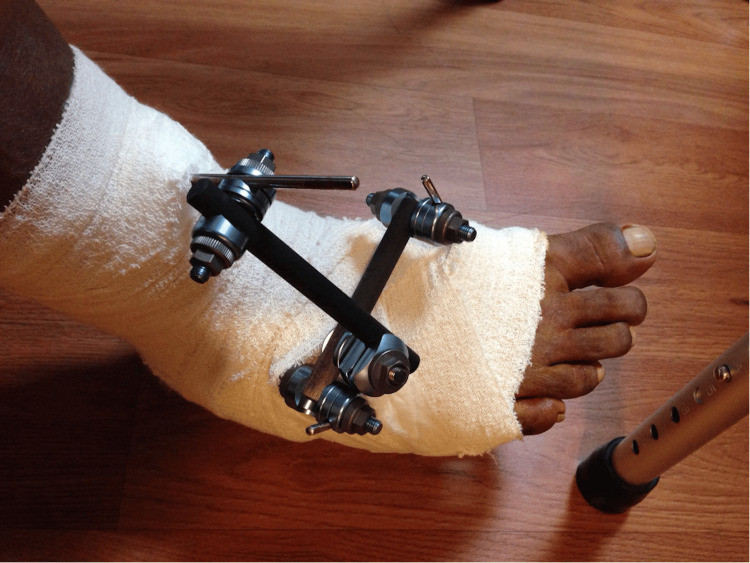
Ankle-spanning external fixator was put on for six weeks.

**Figure 21:**
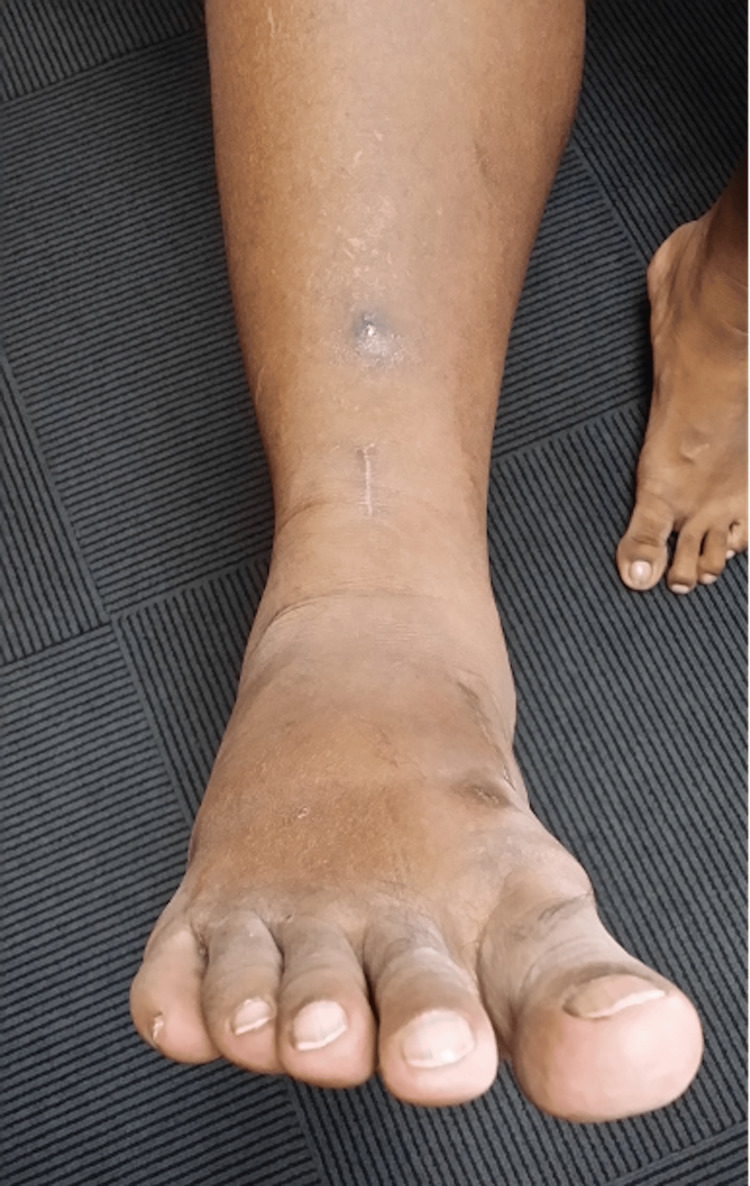
Post-operative photo of the patient in a stable plantigrade position at three months post-surgery.

The anterior tibialis tendon (ATT) was harvested from its insertion over the medial cuneiform and delivered proximally via an anteromedial incision just proximal to the ankle joint. The ATT was then tunnelled subcutaneously distally toward the cuboid and secured to the cuboid using a 2.9 mm suture anchor (Figure 19). He was then placed on an ankle-spanning external fixator for six weeks (Figure 20) with pins in the anterior distal tibia, and first and fifth metatarsal shafts. This is to keep the foot plantigrade for six weeks. We routinely perform this for tendon transfer procedures as it will protect the transfer and allow for easy wound care. In addition, it also allows for a simpler intraoperative process during the positioning of the foot in plantigrade and tensioning of the transferred tendons. The patient was then started on physiotherapy after the external fixator was removed at six weeks. Figure 21 shows the foot in a stable plantigrade position after the external fixator was removed. At three months post-surgery, his foot and ankle remained in a neutral position. It was also in a neutral position on active dorsiflexion of his ankle. At one year post-surgery, his foot and ankle were maintained in a neutral position. His ankle was stable, and he did not complain of any more "floppiness" or tripping.

## Discussion

The lateral compartment of the lower leg compromises the peroneal musculature. The peroneal brevis arises from the mid to lower two-thirds of the fibula, courses behind the lateral malleolus along a fibrocartilaginous retromalleolar groove, passes above the peroneal tubercle, and inserts into the base of the fifth metatarsal base. Its tendon is ovoid in cross-section at the level of the ankle joint and has a low-lying muscle belly, which can extend distal to the superior peroneal retinaculum. The peroneal longus originates from both the tibia and fibula, becomes a rounded tendon, and lies posterior to the peroneal brevis as it courses toward the tip of the lateral malleolus. It then passes inferior to the peroneal tubercle and into the cuboid tunnel before crossing the midfoot plantar and inserts mainly into the medial cuneiform and the base of the first metatarsal.

The blood supply is from the peroneal artery [6]. The avascular portion of the peroneal brevis is at the level of the tip of the lateral malleolus, where the tendon makes a turn. Two vascular zones in the peroneal longus tendon have been elucidated: (1) from the fibular tip to the peroneal tubercle and (2) cuboid tunnel. Consequently, these zones are areas of potential tendinopathy.

There are two accessory muscles of note, namely the peroneal quartus and the peroneal quintus. They can originate from the fibula, peroneal brevis, peroneal longus, peroneal tertius, or a combination of these structures. The peroneal quartus usually inserts into the calcaneus or a slip of the extensor digitorum longus, while the peroneal quintus inserts into the fifth metatarsal. The presence of an accessory muscle can lead to “overcrowding” of the retromalleolar space.

The function of the peroneal musculature is primarily to evert the hindfoot. The peroneal longus contributes to 35% of the total hindfoot eversion strength, while the peroneal brevis provides 28% [7]. They contribute to the stability of the ankle joint and are weak plantar flexors of the ankle. The peroneals can be affected by hindfoot alignment. An excessive hindfoot valgus can cause subfibular impingement of the peroneal tendons, while a varus hindfoot results in increased strain in the tendons.

Chronic peroneal tears are believed to be more common than acute ones, with these tears happening over time after a precipitating event [8,9]. Peroneal brevis tendon tears are more common than peroneal longus tendon tears [10]. The location of tears occurs at regions of hypovascularity under mechanical stress. The peroneal brevis makes a turn at the fibular tip as it courses distally toward the base of the fifth metatarsal bone. This combined with the compression of the tendon between the fibula and the peroneal longus tendon makes it susceptible to fraying or longitudinal tears. Occasionally, an accessory muscle like the peroneal quartus or a low-lying peroneal brevis belly causes “overcrowding” in a limited space, thus increasing the risk of a tendon tear. Similarly, peroneal longus tears occur in areas of regions of high mechanical stress. Brandes and Smith have classified peroneal longus tears into three zones [9]. The first zone refers to the peroneal longus tendon between the tip of the lateral malleolus to the peroneal tubercle, the second zone extends from the lateral trochlear process to the inferior retinaculum, and the last zone stretches from the inferior retinaculum to the cuboid notch. The majority of the peroneal longus tears occur at the last zone where the tendon makes the most acute turn.

In assessing patients with suspected peroneal tendons, the treating clinicians should assess for lateral ankle instability, varus hindfoot deformity, and hypertrophied peroneal tubercle as well as subluxing peroneal tendons with incompetent superior peroneal retinaculum. Weight-bearing radiographs are useful in assessing for any varus hindfoot alignment [7,11]. In addition, the position of the os peroneum should be assessed, as a migration of the os peroneum proximal to the calcaneo-cuboid joint would suggest a tear of the peroneal longus distal to the os peroneum [12]. An MRI scan is a useful modality in assessing for any hypertrophied peroneal tubercle, fluid around the tendon, intra-substance signal changes, and enlargement in the tendons as well as discontinuity of the tendons.

Patients can be treated non-operatively with immobilisation in a boot or a brace for four to eight weeks and physiotherapy with activity moderation and anti-inflammatory medication [13]. Surgery can be considered if surgical management fails. Krause and Brodsky proposed a classification of the peroneal brevis tears according to the extent of cross-sectional area tear involvement, with less than 50% involvement being classified as grade I and more than 50% involvement being considered as grade II. It is suggested that a grade I can be repaired by debridement and tabularization, while a grade II tear should be resected and tenodesed to the adjacent intact peroneal longus tendon. This recommendation was based on the authors’ experience and observation that there was increased mechanical stress occurring in the remnant tendon after more than half was removed [8].

Squires et al. proposed a treatment algorithm that included concomitant tears involving both tendons as well as assessing for functional excursion of intact tendons [14]. Repair was recommended for tendons that appear grossly intact. If one of the tendons is torn and unrepairable, tenodesis to the other functional intact tendon is suggested. If both tendons are torn and unrepairable, a tendon graft or transfer procedure is recommended. 

Seybold et al. reported high patient satisfaction after a single-stage flexor digitorum longus or flexor hallucis longus transfer after concomitant tendon ruptures. Despite significant deficits in strength and balance, patient activity was not altered [15]. In a study of 49 peroneal tears (n = 41 patients), Saxena et al. reported improved American Orthopedic Foot and Ankle Society (AOFAS) scores as well as an average return to activity of 3.49 ± 1.15 months after peroneal tendon surgery. [16]

Taneja et al. found significantly larger peroneal tubercles in patients with infra-malleolar peroneal tendon abnormalities [17]. We routinely remove the peroneal tubercle during surgical repair or tendon grafting of the peroneal tendons. In addition, Taniguchi et al. found an association of increased cavovarus deformity with peroneal tendon tears [18]. We routinely offer concomitant cavus deformity correction for patients with peroneal tendon tears. Peroneal tears were also found to be associated with a concave type of retromalleolar groove, the presence of a peroneal quartus as well as a low-lying peroneal brevis muscle belly [19].

A retrospective study of 40 patients who underwent surgery showed a 10% rate of major complications (persistent symptoms requiring revision surgery as well as 20% of minor complications) [20]. The minor complications included sural neuritis, residual tendonitis, and peroneal tendon subluxation. These resolved eventually and did not affect function.

There has been no general consensus on the optimal period of immobilisation or when the initial range of motion exercises should be started. Rehabilitation should be patient-directed, early protected weight bearing and range of motion exercises should be initiated after a short period of non-weight bearing, and skin stitches should be removed.

## Conclusions

The index of suspicion for diagnosis of peroneal tendon tears should remain high as peroneal tears are often missed. A detailed history and focussed physical examination with the judicious use of imaging will aid in making the correct diagnosis and guide our management. In this paper, we shared the cases of four patients with different combinations of peroneal tears and the different surgical treatments for each of the patients. Management should be individualised and would depend on the extent and chronicity of tendon tear, number of tendons torn, tendon quality as well as associated abnormalities. Treatment may also involve additional bony procedures including cavus deformity correction.
